# Vehicle Tracking for an Evasive Manoeuvres Assistant Using Low-Cost Ultrasonic Sensors

**DOI:** 10.3390/s141222689

**Published:** 2014-11-28

**Authors:** Felipe Jiménez, José E. Naranjo, Oscar Gómez, José J. Anaya

**Affiliations:** University Institute for Automobile Research (INSIA), Ctra. Valencia, Km. 7, Madrid 28031, Spain; E-Mails: joseeugenio.naranjo@upm.es (J.E.N.); labie2.insia@upm.es (O.G.); jjanaya_c@gmail.com (J.J.A.)

**Keywords:** ultrasonic sensor, vehicle positioning, vehicle tracking, speed estimation, collision avoidance

## Abstract

Many driver assistance systems require knowledge of the vehicle environment. As these systems are increasing in complexity and performance, this knowledge of the environment needs to be more complete and reliable, so sensor fusion combining long, medium and short range sensors is now being used. This paper analyzes the feasibility of using ultrasonic sensors for low cost vehicle-positioning and tracking in the lane adjacent to the host vehicle in order to identify free areas around the vehicle and provide information to an automatic avoidance collision system that can perform autonomous braking and lane change manoeuvres. A laser scanner is used for the early detection of obstacles in the direction of travel while two ultrasonic sensors monitor the blind spot of the host vehicle. The results of tests on a test track demonstrate the ability of these sensors to accurately determine the kinematic variables of the obstacles encountered, despite a clear limitation in range.

## 1. Introduction

Many driver assistance systems require some knowledge of the vehicle's surroundings. As these systems are increasing in complexity and performance, this knowledge of the environment needs to be more complete and reliable, establishing an area around the vehicle that must be continually monitored [1,2]. There are several technical solutions for supervising this area. A first classification distinguishes between long- and short-range sensors. In the first group, laser scanners, radar and computer vision are included. In the second group, ultrasonic and capacitive sensors can be highlighted. These sensors also have a wide variety of performance characteristics and their cost can also vary significantly but long-range sensors are usually more expensive and complex than short-range ones. For this reason, when multiple sensors are included in a specific assistance system, the characteristics of each one should be assessed in order to choose those that provide a satisfactory solution without increasing the price of the system unnecessarily.

A collision avoidance system equipped with a laser scanner on the front of the vehicle is described in [3]. This system can detect obstacles and take actions automatically to stop or avoid a collision, assessing whether there are other vehicles in the opposite direction in the adjacent lane. High performance characteristics are required for this sensor because reliable and accurate data are essential for performing safe autonomous manoeuvres. However, this system has the limitation of not taking into account any obstacles in the adjacent lane that are circulating in the same direction as the host vehicle. In particular, the most critical situation in terms of safety would be the case where the system executes a lane change manoeuvre to avoid an obstacle and simultaneously another vehicle is overtaking the vehicle along the adjacent lane. To avoid this situation, the system needs to monitor the adjacent lane in the same direction in which the host vehicle is moving. Obviously, the use of high-performance sensors such as laser scanners used in the detection at the front is a feasible and reliable solution. However, the cost of the system would increase greatly while the features of the sensor exceed the needs of the application. Therefore, in this paper the feasibility of using low-cost ultrasonic sensors is analyzed to detect vehicles in the blind spot to give information on the kinematics of those vehicles to the collision avoidance system that can perform evasive manoeuvres. This monitoring goes beyond current simple detection systems because it is now necessary to know the relative speed of the obstacles. The application scope must be analyzed given the maximum range of these sensors.

The paper is organized as follows: firstly, Section 2 presents some related work on the use of different sensors for supporting driver assistance systems. Then, Section 3 describes the system layout of the collision avoidance system based on the one presented in [3]. Section 4 describes the method for estimating relative speed using ultrasonic sensors and Section 5 includes the selection of the sensors and the performance tests. Finally, Section 6 discusses the limitations of using this sensor system taking into account the decision conditions managed by the avoidance system, while Section 7 presents the main conclusions.

## 2. Related Work

In order to supervise the surroundings of a vehicle, there are several sensors that allow monitoring the environment. The most commonly used are laser scanners [4–6], radar [7–9] and computer vision [10–13]. Different studies compare the advantages and limitations of these technologies [14]. They clearly show the great potential of computer vision for reconstructing a model of the environment, although problems arise in adverse weather conditions and in environments with changing lighting conditions. Furthermore, the high computational cost is a relevant drawback. On the other hand, the measuring range of 77 GHz microwave radar and some laser scanners offer values that are similar in distance and reach more than 200 m. This is not so with the 24 GHz radar which generally operates at shorter distances. However, the laser scanner shows great versatility for working over long and short distances with the same device. The main drawback of radar regarding its measurement field is its limited lateral vision and this fact limits the type of applications these sensors can be used for. Under conditions of fog, rain or heavy snow, radar usually has no problem in detection but the laser scanner is affected by weather conditions since the particles can cause the beam to reflect and disperse, which can attenuate the signal or provide false echoes [15]. These problems have been avoided by increasing the number of horizontal scan planes and/or including filtering algorithms [16]. Finally, it should be noted that, bearing in mind the parameters associated with the distances and type of the objects detected, the analyses show computer vision and the laser scanner to be more versatile tools [17].

Numerous studies indicate that if a complete representation of the environment or an increase in the confidence level in detection is required, it is essential to use sensor fusion from multiple information sources [18–21]. This sensor fusion supports safety applications such as Adaptive Cruise Control based on any of the three technologies mentioned [22,23], pre-crash collision avoidance systems integrating computer vision and laser [24] and autonomous driving systems merging several technologies [22,25,26], *etc.*

On the other hand, other simpler applications such as parking assistants [27–29], finding free parking spots [30,31] or blind spot monitoring, particularly when driving in urban areas [32–35], rely on lower performance and less complex sensors such as ultrasonic sensors whose cost is usually significantly lower than the previously mentioned sensors. However, in this case, their use is usually limited to the detection of obstacles, but they are not of use for tracking and analysing the kinematic characteristics of these obstacles. For example, the sensors used for parking assistant simply warn the driver of the remaining distance to some obstacle. In the case of blind spot monitoring, the system is again limited to alerting the driver of the presence or absence of other vehicles in a specific area around the host vehicle. Other more demanding applications such as adaptive cruise control for urban areas have been based on ultrasonic sensors [36]. Finally, work based on the installation of ultrasonic sensors on a roadside pole to detect vehicles on the road is reported in [37]. Other research using ultrasonic sensors in the automobile field can be found in [38,39]. It is even usual to find research that combines ultrasonic sensors for near range detection and laser scanner [40] or radar [41] for long range detection.

## 3. System Layout

**Figure 1.**
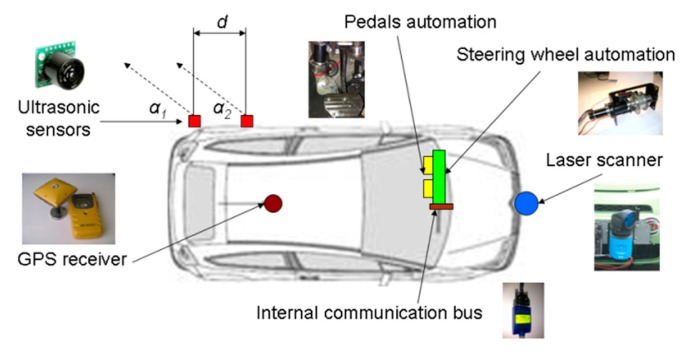
Environment detection sensors embedded in the system.

The detection system presented in [3] is complemented by ultrasonic sensors placed on the rear-side part of the vehicle (Figure 1) in order to complete the vehicle surroundings supervision and detect any vehicle circulating in the blind spot on the adjacent lane.

Thus, the primary environment detection sensor consists of the laser scanner on the front. Specifically, the Sick LRS 1000 sensor is used, whose performance with revised obstacle identification algorithms has been satisfactory proven in [24,42].

Moreover, monitoring the adjacent lane is performed by using two low-cost ultrasonic sensors. The parameters of these sensors are three: distance *d* between them and the angle formed by the beam centre line of each detection sensor and the vehicle side line, *α_1_* and *α_2_*. It should be noted that sensor 1 must be located as near as possible to the rear part of the vehicle in order to achieve the detection of a vehicle circulating on the adjacent lane as soon as possible. Likewise, the small distances *d* and angles should be selected, with the restriction that *α_1_* ≤ *α_2_*.

Finally, in the same way as the case described in [3], the system requires measuring the vehicle speed acquired from the internal communication bus, and positioning it on a digital map, provided by a Topcon DGPS RTK GB-300 receiver [43]. The vehicle steering and speed (acceleration and brake pedals) automation is described in detail in [44].

## 4. Method for Estimating Relative Speed Using Ultrasonic Sensors

### 4.1. Method

**Figure 2.**
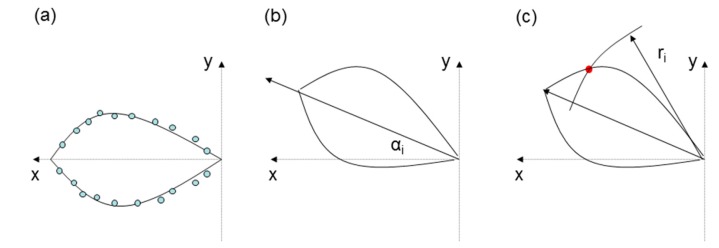
(**a**) Sensor beam fitting; (**b**) Rotation of the sensor beam; (**c**) Identification of the point detected by the ultrasonic sensor.

Given the configuration of the ultrasonic sensors shown in Figure 1, the relative speed between the host vehicle and a vehicle moving along the adjacent lane is determined considering the elapsed time between the detection times of the two sensors. In the simplest situation, the distance between the two detections can be approximated by *d*, although in the case of *α_1_* ≠ *α_2_* or when the path of the two vehicles is not perfectly parallel, this estimation is incorrect. For more accurate results, the point of the sensor beam in which the obstacle is located must be identified. Thus, under the hypothesis that the first point of the vehicle detected by the two sensors of the host vehicle is the same, the following steps are followed in the calculation of the relative speed:

(a) Characterization of the sensor beam.
(b) Curve-fitting of the sensor beam. In general, this curve-fitting is possible using second-degree polynomials following Equation (1) (Figure 2a):
  (1) $$y=A\cdot x^{2}+B\cdot x$$
(c) The beam is rotated the orientation angle *α_i_* around the coordinates origin (Figure 2b). The relationship between the old and new coordinates is given by the well-known Equation (2):
  (2) $$\left(\begin{array}{c} x'\\ y' \end{array}\right)=\left(\begin{array}{cc} \cos\alpha_{i} & -\sin\alpha_{i}\\ \sin\alpha_{i} & \cos\alpha_{i} \end{array}\right)\cdot \left(\begin{array}{c} x\\ y \end{array}\right)$$
  Thus, after some calculations, the new form of the parabola in provided by Equation (3):
  (3) $$y^{\prime }=\frac{-B'\pm \sqrt{B{'}^{2}-4A'C'}}{2A'}$$
  where:
  (4) $$A'=A\cdot \sin^{2}\alpha$$
  (5) B'=2A⋅x'⋅cosα⋅sinα+B⋅sinα−cosα
  (6) $$C'=A\cdot x{'}^{2}\cdot \cos^{2}\alpha +B\cdot x'\cdot \cos\alpha +x'\cdot \sin\alpha$$
(d) Identification of the detection points using the distances *r_1_* and *r_2_* of the first detected point of the vehicle moving along the lane adjacent to both sensors (Figure 2c). This intersection is located at the intersection of the beam sensor and the circumference whose centre is located at the sensor position and the radius is the distance detected by it.
(e) Relative speed (*v_x_, v_y_*) calculation considering the coordinates of the detection points of sensors 1 (*x_1_, y_1_*) and 2 (*x_2_, y_2_*) and the elapsed time between the detection times of the two sensors *T* using Equation (7):
  (7) $$\begin{array}{cc} v_{x}=\frac{x_{2}-x_{1}}{T} & v_{y}=\frac{y_{2}-y_{1}}{T} \end{array}$$

### 4.2. Acquisition Frequency Restrictions

Since the relative speed estimation is based on the detection of one point by each sensor, the frequency of the data acquisition should be high enough to ensure that it can detect one preset speed *v_max_* and that the uncertainty in the measurement time at which the first point of the obstacle is detected is sufficiently low. Specifically, the first requirement dictates that the sampling frequency *f* and the time between two samples *Δt* follow Equation (8):

(8) $$\frac{1}{f}=\Delta t\leq \frac{d}{v_{\max}}$$

Moreover, the resolution regarding the estimation of the relative speed depends on the number of sampling cycles between the detection of the first sensor and the second, and is given by:

(9) $$\text{Speed resolution}=\frac{d}{n\cdot \left(n+1\right)\cdot \Delta t}$$

where *n* is the number of cycles between one detection and another. Thus, the worst resolution occurs when sensor 2 detects the obstacle. In the following sample sensor 1 (*n* = 1) has detected the obstacle, since in this case the speed estimation passes from *v*(*n* = 1) to *v*(*n* = 2) = *v*(*n* = 1)/2.

## 5. Performance Tests of the Ultrasonic Sensors

### 5.1. Ultrasonic Sensors Selection

Table 1 contains the main features of the ultrasonic sensors considered. From these data, the LV-EZ3 sensor is the one that provides greater benefits, as the range, even though limited, is the longest of the sensors considered and, additionally, the sampling frequency exceeds 250 Hz, specified in the section above.

### 5.2. Sensors Configuration and Layout

**Figure 3.**
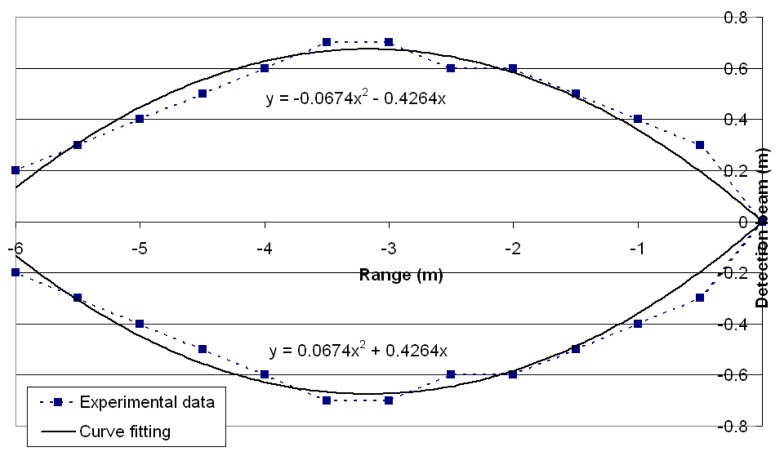
Experimental sensor beam.

Prior to their final assembly the sensors were tested to characterize their operation under similar conditions to those of the subsequent application. Firstly, Figure 3 shows the experimentally measured sensor beam and the curve-fitting using a second degree polynomial.

In addition, several tests were conducted on different configurations of sensors varying *d* and *α_i_* with the purpose of identifying the most convenient configuration. The following conclusions have been reached:

(1) There is a minimum value for the angle *α_i_* below which an obstacle detection of the characteristics of a vehicle circulating in the adjacent lane would not be reliable and there would be a high probability of false negative signals occurring. Several tests were performed with angle values ranging between 10° and 50°, with 5° increments. The limit value that provides reliable obstacle identification was set at 20° because misdetections have not been found up to this value but higher angles provided more than 25% of misdetections, so the detection results lose their reliability. Moreover, it should be noted that this angle is sufficient given the maximum range of the sensor to detect a vehicle travelling in the adjacent lane and assuming standard vehicle dimensions (1.6 m wide or more) and lanes (3.5 m wide or narrower).
(2) When the distance *d* is small and the sensors are working simultaneously, interferences occur in the measurements. Crosstalk is a common problem when multiple ultrasonic sensors are used. In this situation, a sensor receives the signal that another sensor generates and confusing signals are recorded. This fact can lead to false alarms that need to be filtered. Some solutions can be found in the technical literature. In this sense, in [45] a microcontroller-based method to reduce crosstalk between sensors is described, which is achieved by firing each transducer by a pseudorandom number of pulses so that the echo of each transducer can be uniquely identified. For the proposed system, a simpler but reliable and robust solution is used. Thus, the alternate detection of the two sensors is proposed so that once sensor 1 detects an obstacle it is deactivated and sensor 2 will operate until the obstacle is detected and becomes inactive and activates sensor 1. Activation of the sensor by power switching allows only frequencies up to 1.3 Hz, which are insufficient, as mentioned above. However, the enable signal allows switching and acquisition frequencies of 250 Hz. Despite the above, with high frequencies, the switching method does not prevent interference if the distance d is less than 0.3 m.

As above, the final choice of the sensors in the system meets the following characteristics: *d* = 0.3 m, *α_1_* = *α_2_* = 20°. Thus, the sensor detects and estimates the speed of vehicle 3 as far away as possible from the host vehicle.

### 5.3. Vehicle Positioning and Tracking Tests

Finally the assembly of 2 ultrasonic sensors was tested to estimate the overtaking speed of a vehicle simulating the conditions of use of the system. For this purpose, the sensors were located as indicated in Section 4.2. Along the same lines, a Sick LRS 1000 laser scanner was installed in order to compare the speed estimations provided by the two sensors, given the proven reliability of the second [42]. The refresh rate of the laser scanner was 10 Hz with an angular resolution of 0.25°. Moreover, the acquisition frequency of ultrasonic sensors was set to 250 Hz because high frequencies are needed to achieve appropriate resolution results and the correct functioning of these sensors at that frequency had been experimentally verified.

**Figure 4.**
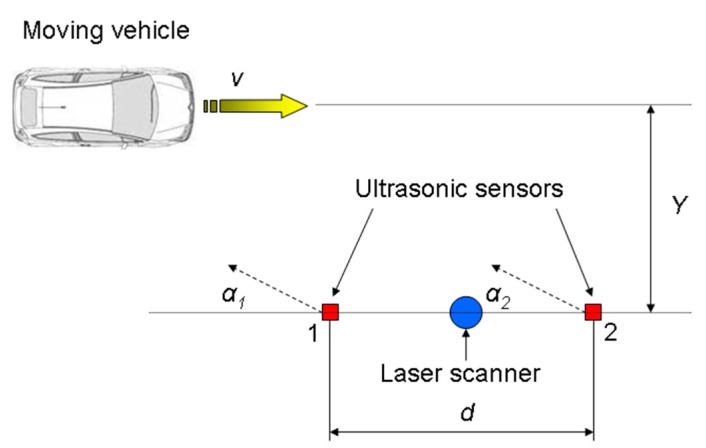
Test layout.

The tests consisted of overtaking a passenger car in a path approximately parallel to the line of sensors at a distance *Y* of approximately 1.5 m from them, at different speeds v between 10 km/h and 40 km/h with increments of 5 km/h, maintained by the vehicle cruise control (Figure 4), trying to simulate the usual operating conditions of the sensors in the driving assistance system. The cruise control system could not maintain the same speed in every test even when the target value was the same, so 5 repetitions were performed at each speed level.

Table 2 shows the results obtained. Because of the dispersion of the real speed achieved by the vehicle when the target speed was the same, average values are shown, but it should be noted that the dispersion is quite narrow so the calculated errors can be considered representative of each speed level. The laser scanner provides very similar results to the real speed measurements from the vehicle communication bus, as already proven in tests in more complex configurations [3]. Moreover, the measurement procedure with the ultrasonic sensors has also provided good results with very small errors even at high speeds (the maximum error is 2.1% detected when the target speed was set at 40 km/h). It should be noted that, because the system measures relative speeds, since detecting relative speeds above those tested is not usual, the usual range of application of the sensor in the detection system has been properly covered. Thus, the ability of the pair of ultrasonic sensors to position and track other vehicles during overtaking manoeuvres along the adjacent lane has been verified with quite satisfactory results.

## 6. Discussion of Feasibility of the Proposed Solution for an Evasive Manoeuvres Assistant System

### 6.1. Driving Scenarios Considered by the Evasive Manoeuvres Assistant System

**Figure 5.**
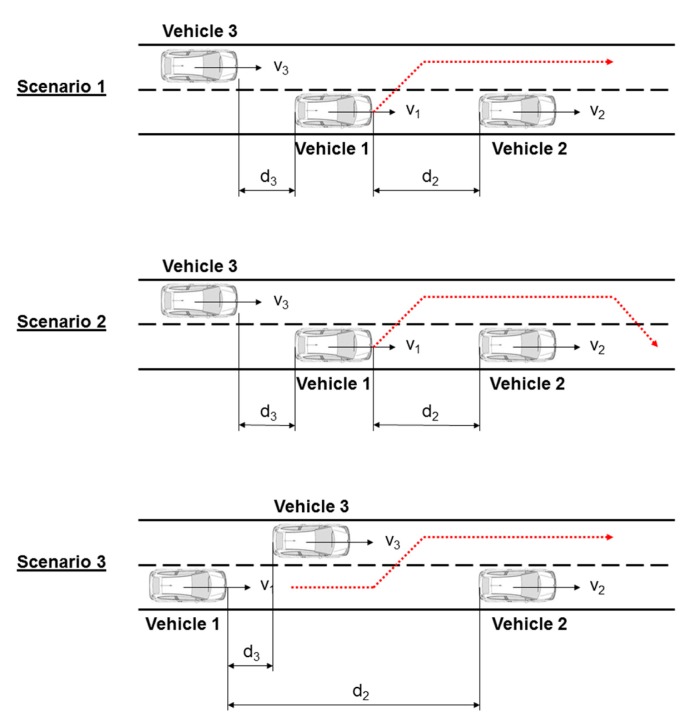
Additional study cases included, using a more complete understanding of the environment.

As stated above, this article is intended to overcome the shortcomings of the environment detection system described in [3] using low-cost solutions, thereby covering a wider range of situations in the decision module of the collision avoidance system. Thus, another three possible study cases have been added to the cases already covered in [3] (Figure 5).

In these, vehicle 1 is travelling at a speed *v_1_* greater than that of vehicle 2 *v_2_* that precedes it in the lane, so vehicle 1 can either choose to perform a braking manoeuvre to adapt the speed or an overtaking manoeuvre. In the previously raised scenarios, vehicle 3, which may prevent overtaking manoeuvres, is moving in the opposite direction along the adjacent lane and, therefore, could be detected by the same sensor responsible for detecting vehicle 2. In these new scenarios, vehicle 3 is moving in the same direction, it is overtaking vehicle 1 (*v_3_* > *v_1_*) or it is being passed by the latter (*v_3_* < *v_1_*) depending on their relative speed.

The decision module system that chooses braking and/or evasive manoeuvres must evaluate in which conditions vehicle 1 can perform these manoeuvres safely to avoid a collision with vehicle 2.

#### 6.1.1. Condition for Safe Braking

The distance between vehicles 1 and 2 should be larger than the value provided by Equation (10) so the speed can be adapted from *v_1_* to *v_2_*:

(10) $$d_{2}\geq \left(v_{1}-v_{2}\right)\cdot t_{r1}+\frac{{\left(v_{1}-v_{2}\right)}^{2}}{2a_{1}}$$

where *t_r1_* is the reaction time between when vehicle 2 is detected and the braking process starts and *a_1_* is the deceleration of vehicle 1.

#### 6.1.2. Evasive Manoeuvre (Scenario 1)

In order for the evasive manoeuvre to be possible, the distance between vehicles 1 and 2 should comply with Equation (11):

(11) $$d_{2}\geq \left(v_{1}-v_{2}\right)\cdot t_{LC}$$

where *t_LC_* is the time required to perform a simple lane change.

Furthermore, in this scenario, vehicle 3 should adapt its speed to vehicle 1's speed before crashing. For this reason, Equation (12) should be verified:

(12) $$d_{3}\geq \left(v_{3}-v_{1}\right)\cdot t_{r3}+\frac{{\left(v_{3}-v_{1}\right)}^{2}}{2a_{3}}$$

where *t_r3_* is the reaction time between when vehicle 1 is detected, and vehicle 3 starts braking and *a_3_* is the deceleration of vehicle 3.

#### 6.1.3. Evasive Manoeuvre (Scenario 2)

In this case, Equation (2) should be verified. Apart from that, depending on the value of d_3_, different scenarios can occur:

(a) Vehicle 3 should decelerate and reach *v_1_*
(b) Vehicle 3 should decelerate and reach a speed $v_{3}^{'}>v_{1}$
(c) Vehicle 3 should not modify its speed

Scenario 2a is the most critical one because it implies a minimum distance *d_3_* below which a collision between vehicles 1 and 3 cannot be avoided. This limit is provided by Equation (12).

Scenario 2c implies that vehicle 1 can perform the overtaking manoeuvre before vehicle 3 collides with it when the latter does not modify its speed. This condition can be expressed mathematically by Equation (13):

(13) $$d_{3}\geq \left(v_{3}-v_{1}\right)\cdot \left(\frac{d_{2}+L_{1}+L_{2}}{v_{1}-v_{2}}+t_{LC}\right)$$

where *L_i_* is the length of vehicle *i*.

In any intermediate situation between Equations (12) and (13), vehicle 3 should modify its speed but its final speed v_3_′ can be higher than v_1_. In order to minimize vehicle 3's speed reduction, this final speed should be reached at the end of vehicle 1's manoeuvre, so Equation (14) provides the final speed of vehicle 3:

(14) $$d_{3}=v_{3}\cdot t_{r3}+\frac{v_{3}^{2}-v_{3}^{'2}}{2a_{3}}-v_{1}\cdot \left(\frac{d_{2}+L_{1}+L_{2}}{v_{1}-v_{2}}+t_{LC}\right)$$

It should be noted that making $v_{3}^{'}=v_{1}$ in Equation (14) provides Equation (12). Furthermore, if Equation (13) is verified, Equation (14) has no sense, because vehicle 3 should not modify its speed and it can maintain *v_3_*. Furthermore, Equations (13) and (14) are not applicable when *v_1_* = *v_2_* because overtaking manoeuvres are not possible in this case.

#### 6.1.4. Evasive Manoeuvre (Scenario 3)

In this case, vehicle 1 should wait to start its manoeuvre until the front part of vehicle 3 reaches the rear part of vehicle 1. Then, the evasive manoeuvre can start and the distance between vehicles 1 and 2 at that moment needs to be large enough to perform the lane change. This condition is provided by Equation (15):

(15) $$d_{2}-v_{1}\cdot \frac{d_{3}+L_{1}+L_{3}}{v_{1}-v_{3}}\geq \left(v_{1}-v_{2}\right)\cdot t_{LC}$$

It should be noted that the overtaking manoeuvre is not possible when *v_1_* = *v_3_*, so Equation (15) is not applicable in this situation.

### 6.2. Discussion of the Range of Application

The major limitation of the detection system lies in the range of the sensors [46]. The most critical situation is contained in the condition established in scenarios 1 and 2a by Equation (12) because this equation establishes the limit for not generating an accident between vehicles 1 and 3 while the former avoids vehicle 2 with an evasive manoeuvre. In this case, it can be seen that the relative speed between vehicles 1 and 3 cannot exceed a certain value. The larger distance *d_3_* that the ultrasonic sensors can detect could be approximated by Equation (16):

(16) $$d_{3}\leq R\cdot \cos\alpha_{2}-d$$

where *R* denotes the maximum range of the sensor. Then, using Equation (12), the maximum relative speed between vehicles 1 and 3 is given by Equation (17):

(17) $$\left(v_{3}-v_{1}\right)\leq a_{3}\cdot \left(\sqrt{t_{r}^{2}+2\cdot d_{3}/a_{3}}-t_{r}\right)$$

For example, if a deceleration level of 4 m/s^2^ [47–49] and a reaction time of 1.5 s [50–52] are chosen for vehicle 3 as typical driver values in this type of manoeuvre, the avoidance system implemented in vehicle 1 can only rely on the ultrasonic sensors speed estimation if *v_3_* − *v_1_* ≤ 10.3 km/h. In the event that vehicle 3 is also equipped with an automatic avoidance system that could take a quicker decision (*t_r_* = 0.5 s) and brake harder (8 m/s^2^ considering that it is an emergency manoeuvre), relative speeds of 21.9 km/h are admissible. This mean that the evasive manoeuvre can only be considered as a safe option by the decision module of the collision avoidance system described in [43] when the relative speed detected is lower than the previous limits. Even if this fact is a clear limitation, this second scenario still provides a relatively acceptable field of application for the ultrasonic sensor-based solution.

Furthermore, the limited range implies that the lateral distance between the host vehicle and the vehicle on the adjacent lane should not be too large. More specifically, when lateral separation is greater than 2.2 m, vehicle 3 cannot be detected using the selected sensors. In spite of this limitation, the speed calculation algorithm based on two ultrasonic sensors is robust, even when vehicles 1 and 3 do not perform exact parallel paths because the exact point in which vehicle 3 enters each sensor detection beam can be obtained and that information is used for calculating *v_x_* and *v_y_* according to Equation (7).

## 7. Conclusions

This paper has analyzed the feasibility of supplementing the collision avoidance system presented in [3] using a pair of ultrasonic sensors to monitor the blind spot in the adjacent lane and provide more options for automatic actuation in the decision module of the collision avoidance system. The initial system was based only on a frontal laser scanner but the limitations regarding detecting the vehicle surroundings were clear and more sensors were required. The use of ultrasonic sensors in this system exceeds its conventional application, which is limited to presence detection, but never to the estimation of the kinematic characteristics of obstacles. Therefore, the contributions of this paper emerge in the use of ultrasonic sensors to obtain information on the kinematics of obstacles moving around the vehicle.

Satisfactory results have been obtained in speed estimation using two ultrasonic sensors, with a level of accuracy similar to that obtained by the laser scanner, which is completely adequate for the ultimate purpose of the collision avoidance system. However, it should be noted that, although the laser scanner can work at acquisition frequencies of 10 Hz without affecting its accuracy, because of the precise location of the obstacle with high resolution, in the case of ultrasonic sensors, acquisition frequencies of about 250 Hz are required, since it is critical to identify the specific instant at which each sensor detects the first point of the obstacle. The other limitation of the detection system is the limited range of the ultrasonic sensors. More specifically, there are restrictions on the relative speed between vehicles 1 and 3 but, as shown above they are not as limiting as might first appear and the avoidance system can still work properly in a wide range of scenarios.

Finally, it should be noted that the actual detection system is not complete enough to assess the surroundings of the subject vehicle with full reliability. For this reason, a medium-range sensor needs to be included in the rear part of the vehicle. Then, the information that this new sensor would provide would be fused with the detections of the ultrasonic sensors that work correctly in a short range as presented in this paper. One of the main objectives is to obtain a low-cost detection system in which only the front sensor (responsible for the detection of the primary obstacle) would be a high-performance sensor, while the others would complement the information but without significantly increasing the total cost of the system.

## Figures and Tables

**Table 1.** Ultrasonic sensors analysed.

**Type**	**Image**	**Transducers**	**Frequency**	**Range**	**Output**
LV-EZ3	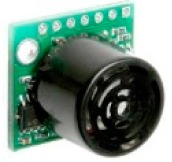	Simple	42 KHz	6.45 m	Analog
SRF02	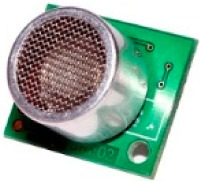	Simple	40 KHz	15 cm–6 m	I2C
SRF08	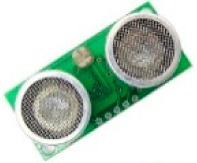	Double	40 KHz	6 m	I2C
SRF10	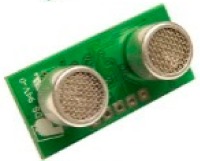	Double	40 KHz	6 m	I2C
SRF235	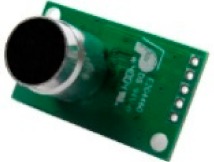	Simple	235 KHz	10 cm–1.2 m	I2C

**Table 2.** Comparison between estimated and measured speeds (average values of five repetitions at each target speed).

**Target Speed (km/h)**	**Real Speed (Internal Communication Bus) (km/h)**	**Speed Calculation (km/h)**		**Error Ultrasonic Sensors** ***vs.*** **Real Speed (%)**

		**Laser Scanner**	**Ultrasonic Sensors**	
10	8.4 ± 0.1	8.4	8.3	−1.2
15	14.9 ± 0.2	14.7	14.8	0.7
20	20.0 ± 0.2	19.8	20.1	1.5
25	25.4 ± 0.3	25.0	25.2	0.8
30	31.5 ± 0.2	30.9	31.3	1.3
35	35.9 ± 0.3	35.4	35.8	1.1
40	44.3 ± 0.2	43.9	44.8	2.1
